# RE: Impact of COVID-19 on a urology residency program

**DOI:** 10.1590/S1677-5538.IBJU.2021.0060

**Published:** 2021-03-05

**Authors:** Ali Atan

**Affiliations:** 1 Gazi University Tip Fakultesi BesevlerAnkara Turkey Gazi University Tip Fakultesi, Besevler, Ankara, Turkey.

*To the editor,*

First of all, I congratulate the authors for their paper titled “Impact of COVID-19 on a urology residency program”. In this paper, the authors stated how the urology residency training program is negatively affected by COVID-19 pandemic.

Medical education has two aspects: Increasing the resident's knowledge of urology and improving their surgical skills. Due to the COVID-19 pandemic, there have been many problems related to core residency training program and residency training methods. While theoretical training is required to increase the knowledge of the residents, practice is necessary to improve their skills. Since theoretical trainings can be maintained via webinars, it can be stated that this aspect of the medical education is less harmed by the pandemic. However, the situation is different for the second aspect since webinars are not appropriate tools to improve surgical expertise, especially surgical skills. Surgical practice is the key element of improving skills as a surgeon and thus, it is crucial for the surgical residents. It is very unfortunate that the surgical residents could not have the opportunity to do surgical practice. As mentioned in this paper (1) and the previous published papers (2, 3) on this topic, the COVID-19 pandemic decreased the surgery volume in urology residency programs.

Reduction of the number of patients admitted to hospitals was an imperative to decrease and prevent the risk of COVID-19 transmission. Therefore, it was recommended that elective surgeries should be postponed or cancelled (4, 5) The EAU published a COVID-19 guideline categorizing the urological procedures in terms of priority. In that guideline, urological procedures were divided as emergent, high-prioritized, intermediate and low-prioritized. According to this categorization, some of urological procedures were found eligible to postpone for more than 6 months (6). As a result of these recommendations of the EAU, there was a significant reduction in the number of the surgical procedures. Inevitably, the chance of urology residents to perform complex surgeries significantly decreased.

There have been many valuable recommendations proposed in order to minimize the negative effects of this decrease in the number of operations made by the urology residents and provide the required quality in medical education. Danilovic et al. recommended simulation laboratory for surgical skills improvement in a dry lab and a wet lab with porcine surgeries as recommended by previous studies (1, 7–9). Another recommendation was to watch high-quality surgical videos to improve surgical skills (10). In my opinion, edited videos may not be as beneficial as expected for a resident to improve his or her skills. Watching unedited original videos would surely be more beneficial because it would allow the residents to see the complications or unwanted situations that may occur during the procedure. Hence, urology residents can find the opportunity to witness the methods of coping with these potential problems and it will certainly be an important winning which they should obtain during their medical education. It will also eliminate the risk of surgical procedures to seem very effortless and simple. Edited videos may cause surgical procedures to be perceived mistakenly as very easy by the residents (11).

According to the opinion by Esperto et al., hospitals should be divided into COVID-19 free and COVID-19 hospitals to continue urological surgeries. The aim of this opinion is to prevent any possible COVID-19 positive patient to access a COVID-19 free hospital. The authors mentioned that this will allow urological surgical activity to carry on, reducing the number of postponed cases (4).

In these difficult times, it would be appropriate to carefully evaluate all these valuable recommendations and provide solutions by taking necessary precautions in terms of both protecting the quality of the education and the health of residents.
